# Effects of maternal gestational diet, with or without methionine, on muscle transcriptome of *Bos indicus*-influenced beef calves following a vaccine-induced immunological challenge

**DOI:** 10.1371/journal.pone.0253810

**Published:** 2021-06-24

**Authors:** Elizabeth A. Palmer, Francisco Peñagaricano, Marcelo Vedovatto, Rhaiza A. Oliveira, Sena L. Field, Jimena Laporta, Philipe Moriel

**Affiliations:** 1 IFAS–Range Cattle Research and Education Center, University of Florida, Ona, FL, United States of America; 2 Department of Animal and Dairy Sciences, University of Wisconsin, Madison, WI, United States of America; 3 Unidade Universitária de Aquidauana, Universidade Estadual de Mato Grosso do Sul, Aquidauana, MS, Brazil; University of Illinois, UNITED STATES

## Abstract

Maternal nutrition during gestation can cause epigenetic effects that translate to alterations in gene expression in offspring. This 2-year study employed RNA-sequencing technology to evaluate the pre- and post-vaccination muscle transcriptome of early-weaned *Bos indicus*-influenced beef calves born from dams offered different supplementation strategies from 57 ± 5 d prepartum until 17 ± 5 d postpartum. Seventy-two Brangus heifers (36 heifers/yr) were stratified by body weight and body condition score and assigned to bahiagrass pastures (3 heifers/pasture/yr). Treatments were randomly assigned to pastures and consisted of (i) no pre- or postpartum supplementation (**NOSUP**), (ii) pre- and postpartum supplementation of protein and energy using 7.2 kg of dry matter/heifer/wk of molasses + urea (**MOL**), or (iii) MOL fortified with 105 g/heifer/wk of methionine hydroxy analog (**MOLMET**). Calves were weaned on d 147 of the study. On d 154, 24 calves/yr (8 calves/treatment) were randomly selected and individually limit-fed a high-concentrate diet until d 201. Calves were vaccinated on d 160. Muscle biopsies were collected from the same calves (4 calves/treatment/day/yr) on d 154 (pre-vaccination) and 201 (post-vaccination) for gene expression analysis using RNA sequencing. Molasses maternal supplementation led to a downregulation of genes associated with muscle cell differentiation and development along with intracellular signaling pathways (e.g., Wnt and TGF-β signaling pathway) compared to no maternal supplementation. Maternal fortification with methionine altered functional gene-sets involved in amino acid transport and metabolism and the one-carbon cycle. In addition, muscle transcriptome was impacted by vaccination with a total of 2,396 differentially expressed genes (FDR ≤ 0.05) on d 201 vs. d 154. Genes involved in cell cycle progression, extracellular matrix, and collagen formation were upregulated after vaccination. This study demonstrated that maternal supplementation of energy and protein, with or without, methionine has long-term implications on the muscle transcriptome of offspring and potentially influence postnatal muscle development.

## Introduction

During the last trimester of gestation, the protein, energy, and amino acid requirements of beef cows increase in order to meet the demand of the growing fetus [1]. Previous studies observed that supplemental protein during the last trimester of gestation increased calf weaning body weight, carcass weight, and marbling scores at slaughter [2–4]. Postnatal muscle development is highly dependent on in utero formation of muscle fibers as the number of muscle cells is set at birth [5]. In the beef cattle fetus, secondary myogenesis predominately occurs during the second trimester of gestation [6]. During the last trimester of gestation, muscle tissue growth continues primarily through muscle hypertrophy and adipogenesis is initiated [6]. Modifications to the maternal diet, either nutrient excess or restriction, during mid- to late gestation can modulate the expression of genes involved in myogenesis and adipogenesis of the muscle [7, 8]. Nutrient restriction during the last 40 d of gestation alters the muscle transcriptome of beef calves at weaning [9], indicating that maternal nutrition during late gestation can program long-term gene expression in the offspring.

Methionine is a limiting amino acid for both multiparous and primiparous beef cows grazing low-quality forages during the last trimester of gestation [10, 11]. Methionine is an essential amino acid that has many important functions within the body, including protein synthesis and DNA methylation. Previous work in livestock species has reported considerable changes in offspring DNA methylation when methyl donors are added to the maternal diet [12, 13]. Methionine acts as a precursor for S-adenosylmethionine, a metabolite of the one-carbon cycle [14]. S-adenosylmethionine plays an active role in both DNA and histone methylation which in turn regulates gene transcription [15, 16]. Indeed, maternal supplementation of methionine during the last trimester of gestation influences hepatic gene transcription in dairy calves [17, 18].

The impact of maternal methionine supplementation on calf postnatal growth is controversial. Supplementation with methionine during the last trimester of gestation in dairy cows increased calf birth weights and body weight gain during the first 9 weeks of postnatal life [17, 19, 20]. Nevertheless, supplementing methionine to beef cows during the first or last trimester of gestation did not impact calf postnatal growth [21–24]. The mechanisms eliciting these changes, or lack thereof, are not well understood. Liu and colleagues reported that the muscle transcriptome of beef calves was modified when cows were supplemented with 10 g/d of methionine during periconception and through the first trimester of gestation [13]. However, little information is known on how methionine supplementation during the last trimester of gestation may influence the postnatal muscle transcriptome in beef calves.

Previous work by our research group observed that calves born to *Bos indicus*-influenced cows provided pre- and postpartum supplementation of protein and energy had greater pre- and post-weaning growth performance compared to calves born from non-supplemented cows, whereas the methionine fortification of supplements did not further enhance calf growth [22, 23]. Further, maternal supplementation with energy and protein enhanced the antibody response in beef calves following vaccination [22]. During an immunological challenge, nutrients are shifted towards developing an immune response at the expensive of growth [25]. Sanglard and colleagues identified correlation networks of immune related genes that were upregulated in blood but downregulated in the muscle following vaccination [9], indicating an interaction between muscle and the immune response. Moreover, dietary amino acid requirements are altered during periods of inflammation and stress [26]. In particular, maternal supplementation with methionine during the last trimester of gestation can enhance the innate immune response of calves [27]. We hypothesized that, during an immunological challenge, we would observe differences in gene expression in pathways related to muscle development and growth in calves born to heifers that received pre- and post-partum molasses supplementation. Further, we anticipated that methionine would produce changes in gene expression due to its role as a methyl donor. Therefore, the specific aim of this study was to gain insight into individual genes and pathways influenced in the *Longissimus dorsi* muscle of calves, following an immunological challenge, when their dams were provided pre- and postpartum supplementation with energy and protein, with or without methionine fortification.

## Materials and methods

### Animals and data collection

This 2-yr study was conducted at the University of Florida, Institute of Food and Agricultural Sciences, Range Cattle Research and Education Center (RCREC), Ona, Florida (27°23′N and 81°56′W). All animal procedures described herein were approved by the University of Florida Institute of Animal Care and Use Committee (protocol #201709982). This experiment was part of a larger study that investigated the effects of pre- and postpartum supplementation with molasses + urea, with or without methionine hydroxy analog fortification, on the physiology and growth performance of beef heifers and their offspring [22]. Briefly, on d 0 of each year, 36 Brahman × Angus heifers (20 to 22 mo. of age) were stratified by initial body weight (396 ± 24.1 kg) and body condition score (**BCS**; 5.67 ± 0.43) and assigned randomly to 1 of 12 bahiagrass (*Paspalum notatum*) pastures (3 heifers/pasture; 1.2 ha/pasture). Treatments were assigned randomly to pastures (4 pastures/treatment) and consisted of (i) no supplementation (**NOSUP**), (ii) supplementation of protein and energy using sugarcane (*Saccharum officinarum*) molasses + urea (**MOL**; 7.2 kg of DM/heifer/wk; Westway Feed Products LLC, Clewiston, FL), or (iii) MOL fortified with 105 g/heifer/wk of methionine hydroxy analog (**MOLMET**; MFP, Novus International Inc., Romance, AR). Molasses + urea supplements were formulated for heifers to gain 0.5 BCS during the last 57 ± 5 d of gestation [1, 22]. Methionine hydroxy analog was offered at the greatest recommended amount by the company for growing beef heifers (15 g/d). Clements et al. reported an increase in plasma methionine precursors in multiparous cows when offered methionine hydroxy analog at a rate of 10 g/d [21]. Moreover, supplementing 15 g/d of _DL_ -methionine during the last 58 ± 1.02 d of gestation successfully increased plasma methionine concentrations in beef heifers [11]. Therefore, treatment supplementation was initiated on d 0 (57 ± 5 d prepartum) and continued until all heifers within each pasture had calved (17 ± 5 d postpartum).

Calves were early-weaned on d 147 of each year (89 ± 5 d of age) and were immediately transferred to a dry-lot pen as a single group. Calves remained in the dry-lot pen until d 154 to allow calves to adjust to a concentrate diet (Purina® Precon® Complete; Land O’Lakes Purina Feed LLC, Gray Summit, MO) and overcome the stress of weaning. On d 154, 24 calves/yr (4 heifer and 4 steer calves/treatment/yr) were selected randomly and transferred to individual concrete, covered pens where they remained until the end of the study on d 201. From d 154 to 201, calves were limit-fed a high-concentrate diet [27.2% crude protein (**CP**) and 75.0% total digestible nutrients (**TDN**) on a dry-matter (**DM**) basis] starting at 2% of shrunk BW and increasing to 3.5% of shrunk BW with concentrate increasing in increments of 0.5% or less. Additionally, calves were offered 1 kg/d of long-stem stargrass (*Cynodon nlemfuensis*) hay (7.3% CP and 53.5% TDN on a DM basis) and a complete salt-based mineral supplement (Cattle Select Essentials Range, Lakeland Animal Nutrition, Lakeland, FL). On d 160, calves were vaccinated against infectious bovine rhinotracheitis, bovine viral diarrhea virus type 1 (**BVDV-1**) and 2, parainfluenza-3 virus (**PI-3**), bovine respiratory syncytial virus, and *Mannheimia haemolytica* (2 mL subcutaneous; Bovi Shield Gold One Shot; Zoetis, Parsippany, NJ), as well as *clostridium* (2 mL subcutaneous; Ultrabac 7, Zoetis) and administered an oral anthelmintic (5 mg/kg of BW; Safe-guard, Merck Animal Health, Summit, NJ) to protect against internal parasites. On d 188, calves were administered a booster vaccination of Bovi Shield Gold One Shot (2 mL subcutaneous; Zoetis) and Ultrabac 7 (2 mL subcutaneous; Zoetis). This vaccination protocol was utilized to stimulate an immune response [28–30]. Additional details on management, diets and the complete nutrient analysis have been previously reported [22].

On d 154 and 201 of yr 1 and 2, *Longissimus dorsi* muscle samples were collected from 12 randomly selected calves (4 calves/treatment). Muscle biopsies were collected on both heifers (n = 6; 2 heifers/treatment) and steers (n = 6; 2 steers/treatment) in year 1 and only steers in year 2, respectively. The same 12 calves were biopsied at both timepoints. Muscle biopsies were conducted by a single, trained individual. Approximately 50 mg of muscle samples were collected from the *Longissimus dorsi* muscle located above the 11^th^ and 12^th^ rib using a Tru-Cut biopsy needle (14-gauge × 15 cm; CareFusion; Becton Dickinson, Franklin Lakes, NJ). Muscle biopsies were always conducted on the right side of the calf following a 12-h period of food and water withdrawal. Immediately after sample collection, muscle samples were contained in aluminum foil and snap-frozen with liquid nitrogen. Muscle samples were stored at -80°C until RNA extraction.

### RNA extraction, library preparation and sequencing

Muscle tissue was homogenized in 400 μl of TRIzol™ Reagent (Invitrogen, Carlsbad, CA) with 3.0 mm zirconium beads (#D1032-30; Thomas Scientific, Swedesboro, NJ) using a high-throughput bead tissue homogenizer (Precellys 24; Bertin Technologies SAS, Montigny-le-Bretonneux, France). Tissues were homogenized for 10 s at 5000 rpm, set on ice for 30 s, and then homogenized again for 10 s at 5000 rpm. Following muscle tissue homogenization, 100 μl of chloroform was added to the sample and centrifuged at 12,000 × g for 15 min at 4°C. The aqueous layer was isolated and combined with 70% ethanol at a 1:1 ratio before RNA was extracted and purified using the RNeasy Mini Kit (catalog #74104; Qiagen, Valencia, CA). The RNA concentration and quality were determined using the Agilent 2100 bioanalyzer (Agilent Technologies, Santa Clara, CA). Following RNA quantification, 3 RNA samples (n = 1 and 2 for NOSUPP and MOL, respectively) were not included in subsequent library preparation due to low concentrations of RNA (RNA concentration < 0.2 μg). The remaining 45 samples used for library construction had an RNA integrity number of 6.1 or greater. Library construction was conducted using the commercial kit NEBNext Ultra II RNA Library Prep by Illumina (#E7775; New England BioLabs, Ipswich, MA). Individual library concentrations were initially assessed using the Qubit 2.0 (ThermoFisher, Invitrogen, Grand Island, NY), sized using the Agilent 2100 bioanalyzer (Agilent Technologies), and quantified with qPCR. After passing quality control, 45 individual barcoded libraries were pooled at equal molar concentrations. Lastly, sequencing was performed using the Illumina NovaSeq 6000 platform (San Diego, CA) and produced paired-end 150 base-pair reads. Library construction and sequencing was performed by Novogene Inc. (Sacramento, CA).

### RNA-seq analysis: Quality control, read mapping, and gene expression estimation

Quality of the reads was evaluated before and after trimming using FastQC (version 0.11.7, Babraham Bioinformatics, UK). Trimming was performed using Trim Galore (version 0.4.4, Babraham Bioinformatics, UK) with the following parameters:—*paired*,—*quality* 20,—*length* 50,—*clip_R1* 15,—*clip_R2* 15,—*three_prime_clip_R1* 5, and—*three_prime_clip_R2* 5. After processing, reads were mapped to the latest bovine reference genome (ARS-UCD1.2) using Hisat2 (v2.1.0) [31]. Finally, the number of reads that mapped to each annotated gene in the bovine annotation file (GTF file) was obtained using the python script *htseq-count* (v0.6.1p1) using the option *intersection-nonempty* [32]. One sample was removed due to low quality reads. Therefore, 44 samples (n = 14, 14, and 16 for NOSUP, MOL, and MOLMET, respectively) were used for downstream analysis of differentially expressed genes and gene-set enrichment analysis.

### RNA-seq analysis: Differential expression analysis

Genes with counts per million ≤ 1 were removed from the raw expression data and not included in the statistical analysis. Gene counts were normalized across biological replicates using the method trimmed mean of M-values implemented in the *R* package edgeR [33]. The expression of each gene (*n* = 13,010) was evaluated using the following generalized linear mixed model:

log(ge)=block+trt+time+trt×time+calf(trt)+e

where *ge* represents the normalized gene expression of the gene under consideration, *block* represents the year of trial (2 levels), *trt* represents the treatment effect (3 levels, NOSUP, MOL, and MOLMET), *time* represents the time effect (2 levels, d 154 and d 201), *trt*×*time* represents the interaction effect treatment-by-time, and *calf*(*trt*) represents the random effect of the calf nested within treatment. Two orthogonal contrasts were evaluated: (1) the effect of supplementation (MOL + MOLMET vs NOSUP) and (2) the effect of methionine (MOLMET vs MOL). Kenward-Roger method was used to calculate the approximate denominator degrees of freedom for the *F* tests. The effect calf nested within treatment was used as the error term for testing the effects of treatment. Finally, *P*-values were adjusted for multiple testing using the false discovery rate (**FDR**) method [34]. A heatmap [35] was generated to display the difference (FDR ≤ 0.05 and a log_2_ fold change (log_2_FC) ≥ |1|) in gene expression on d 201 vs. d 154.

### Gene-set enrichment analysis

The enrichment of Gene Ontology (**GO**) terms and Kyoto Encyclopedia of Genes and Genomes (**KEGG**) pathways with differently expressed genes (**DEG**) were analyzed using Fisher’s exact test. Genes were assigned to GO terms using the function *getBM* from the *R* package biomaRt (v 2.36.1) and KEGG terms were assigned using the *R* package EnrichKit. The Fisher’s exact test, a test of proportions based on the cumulative hypergeometric distribution and commonly used to evaluate 2 × 2 contingency tables, was implemented using the function *fisher*.*test* in the *R* software. Differentially expressed genes for enrichment analysis were designated at *P* < 0.05 for the effect of maternal methionine fortification vs. no methionine fortification, *P* < 0.025 for the effect of maternal supplementation vs. no supplementation, and *P* < 0.0001 for the effect of time. Different thresholds were used to capture more genes for each independent gene set analysis. Functional processes were considered significant when *P* ≤ 0.05. Given that terms are not independent, a classical multiple test approach would be overly conservative, and it was not performed.

## Results

### Mapping summary

Forty-four RNA samples were successfully analyzed from the *Longissimus dorsi* of early weaned *Bos indicus*-influenced calves to determine the effect of maternal supplementation with molasses, with or without methionine fortification, during the last 57 ± 5 d of gestation until 17 ± 5 d postpartum. RNA-sequencing generated approximately 28.9 million paired-end reads per sample with 93% of the total reads successfully mapped to the bovine genome. From those aligned, approximately 91% were mapped to unique regions of the bovine genome. A mapping summary is present in S1 Table. Sequencing data can be accessed through GEO with accession number GSE168091.

### Differential expression in *longissimus dorsi* muscle

A total of 13,010 genes were evaluated for differential expression in the *Longissimus dorsi* muscle of calves for the effect of maternal supplementation with molasses, maternal supplementation of methionine, time of muscle sample collection, and the resulting interaction. No major genes (FDR ≥ 0.25) were identified for the treatment × time interaction; therefore, the main effects will be discussed herein. A full list of DEG for maternal supplementation with protein and energy, maternal supplementation of methionine, and time can be found in S2 Table. Additionally, all significant enrichment GO terms can be found in S3 Table along with the up- and downregulated genes associated with each functional process.

#### Effects of maternal protein and energy supplementation

Pre- and postpartum supplementation of protein and energy did not result in any major DEG (FDR ≥ 0.20) compared to calves born to NOSUP heifers. Consequently, individual genes were investigated at a less stringent threshold to better understand the biological impact of maternal supplementation of protein and energy on muscle transcriptome. Utilizing a less stringent approach (*P* ≤ 0.01), 118 genes were differentially expressed in the muscle of calves as a result of maternal supplementation of protein and energy (S2 Table). Interestingly, all 118 genes identified were downregulated in calves born to heifers that received pre- and postpartum supplementation of protein and energy compared to calves born to NOSUP heifers.

**Fig 1:**
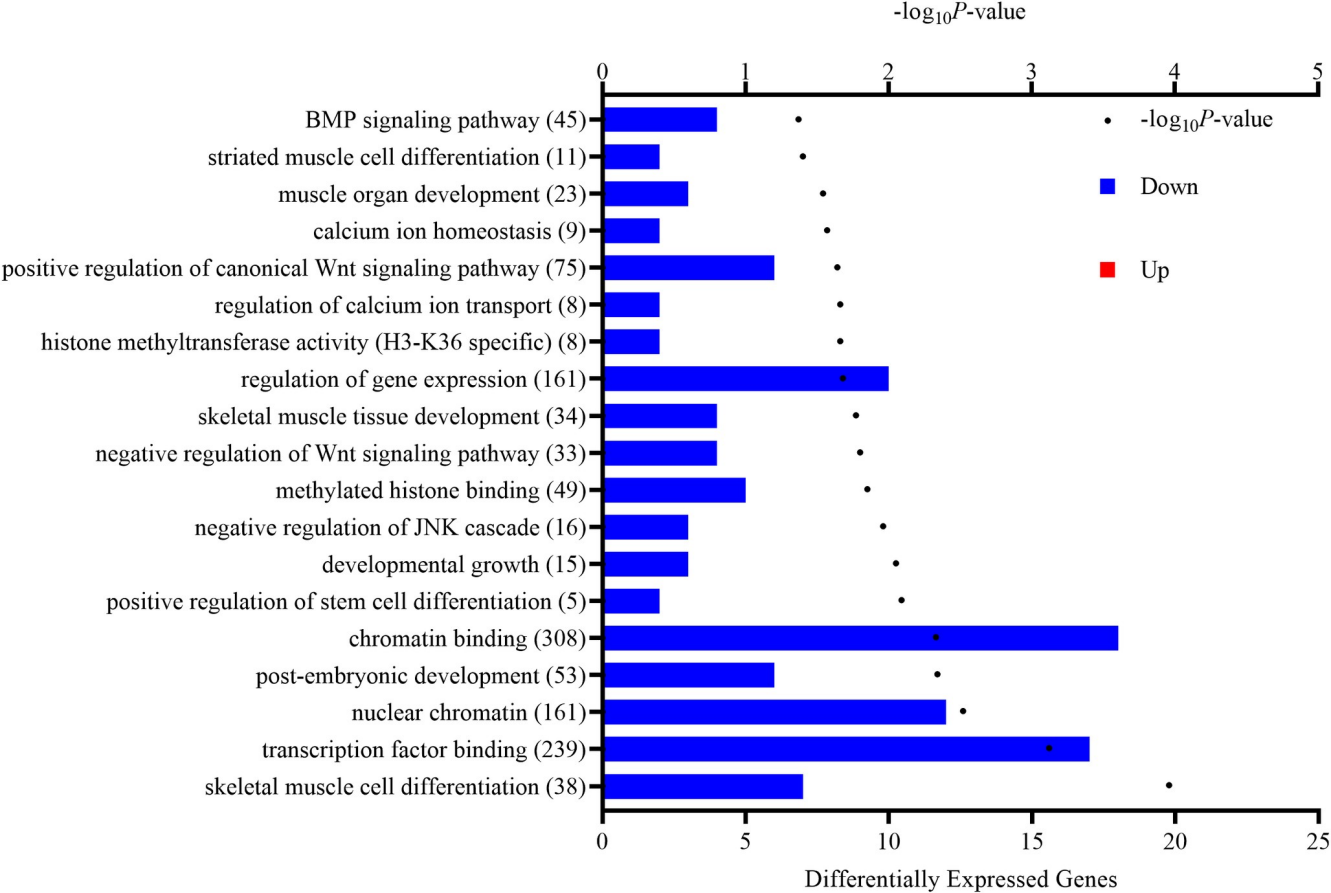
Significantly enriched gene ontology (GO) terms in the *Longissimus dorsi* of early-weaned *Bos indicus*-influenced calves for the effect of maternal supplementation with protein and energy. The number of differentially expressed genes (*P* ≤ 0.025) is reported along the bottom x-axis and is depicted by the bars while the -log_10_*P*-value is reported along the top x-axis and is indicated by the dots. Blue bars indicate downregulated genes while red bars indicate upregulated genes. The number in parentheses is the total number of genes associated with each functional term.

Gene enrichment analysis using DEG (*P* ≤ 0.025; 394 genes) for maternal supplementation of protein and energy identified 10 significant KEGG pathways (Table 1) and 183 significant GO terms (Fig 1). All significantly enriched KEGG pathways and GO terms primarily consisted of genes that were downregulated with protein and energy supplementation. Maternal supplementation of protein and energy influenced various intracellular signaling pathways as indicated by enriched KEGG pathways (e.g., *Hippo signaling pathway*, *TGF-β signaling pathway*, *signaling pathways regulating pluripotency of stems cells*, and *glucagon signaling pathways*) and GO terms (e.g., *negative regulation of Wnt signaling pathway* and *BMP signaling pathway*). Additionally, several GO terms associated with epigenetics were enriched with maternal supplementation of protein and energy such as *histone methyltransferase activity (H3-K36 specific)*, *methylated histone binding* and *chromatin binding*. Gene enrichment analysis also generated GO terms associated with growth and development (e.g., *post-embryonic development*, *developmental growth*, and *anatomical structure development*) and more specifically with muscle development including *muscle organ development*, *skeletal muscle cell differentiation*, and *skeletal muscle tissue development*.

**Table 1:** Significantly enriched Kyoto Encyclopedia of Genes and Genomes (KEGG) pathways in the *Longissimus dorsi* muscle of early-weaned *Bos indicus*-influenced calves for the effect of maternal supplementation of protein and energy^1^.

		Differentially expressed genes^2^		
KEGG term	No. DEG	Upregulated	Downregulated	*P*-value
Hippo signaling pathway	12	-	*BMPR1A*, *FZD7*, *BMP2*, *CCN2*, *SMAD4*, *APC*, *TGFBR2*, *STK3*, *FRMD6*, *TEAD1*, *BIRC2*, *SMAD2*	<0.001
ABC transporters	5	-	*ABCD3*, *ABCC9*, *ABCA1*, *ABCA7*, *LOC51533*	0.001
TGF-beta signaling pathway	8	-	*BMPR1A*, *DCN*, *NEO1*, *BMP2*, *SMAD4*, *ACVR2A*, *TGFBR2*, *SMAD2*	0.001
Signaling pathways regulating pluripotency of stem cells	9	-	*BMPR1A*, *FZD7*, *JAK1*, *PCGF5*, *SMAD4*, *ACVR2A*, *APC*, *MYF5*, *SMAD2*	0.003
Ether lipid metabolism	4	*-*	*CHPT1*, *PLPP1*, *ENPP2*, *PAFAH1B1*	0.006
Th17 cell differentiation	7	-	*NFATC1*, *JAK1*, *FOS*, *SMAD4*, *IL1RAP*, *TGFBR2*, *SMAD2*	0.013
Estrogen signaling pathway	7	-	*CALM2*, *FOS*, *CREB1*, *SOS2*, *ESR1*, *NCOA1*, *GNAS*	0.016
Glucagon signaling pathway	7	-	*PFKFB1*, *CALM2*, *PHKB*, *CREB1*, *PPARGC1A*, *PRKAA2*, *GNAS*	0.018
Osteoclast differentiation	7	-	*NFATC1*, *JAK1*, *FOS*, *CREB1*, *ITGB3*, *TAB2*, *TGFBR2*	0.021
Hippo signaling pathway—multiple species	3	-	*STK3*, *FRMD6*, *TEAD1*	0.036

^1^Treatments consisted of: no supplementation (**NOSUP**); supplementation of protein and energy using sugarcane molasses + urea (**MOL**; 7.2 kg of DM/heifer/wk; Westway Feed Products LLC, Clewiston, FL); or MOL fortified with 105 g/heifer/wk of methionine hydroxy analog (**MOLMET**; MFP, Novus International Inc., Romance, AR). Treatments were initiated on d 0 (57 ± 5 d prepartum) and continued until all heifers within a pasture had calved (17 ± 5 d postpartum).

^2^Threshold for differentially expressed genes was set at *P* ≤ 0.025

#### Effects of maternal methionine supplementation

Pre- and postpartum diet fortification with methionine did not lead to any major DEG (FDR ≥ 0.20) in the muscle tissue. Therefore, individual genes were considered at a less stringent threshold (*P* ≤ 0.01) in order to explore the biological impacts of methionine supplementation on the muscle transcriptome of calves. Under this approach, 26 genes were differentially expressed in the muscle of calves born to dams on MOLMET vs. MOL treatments. Of the 26 genes, 15 genes were downregulated, and 11 genes were upregulated in MOLMET calves vs. MOL calves (S2 Table).

**Fig 2:**
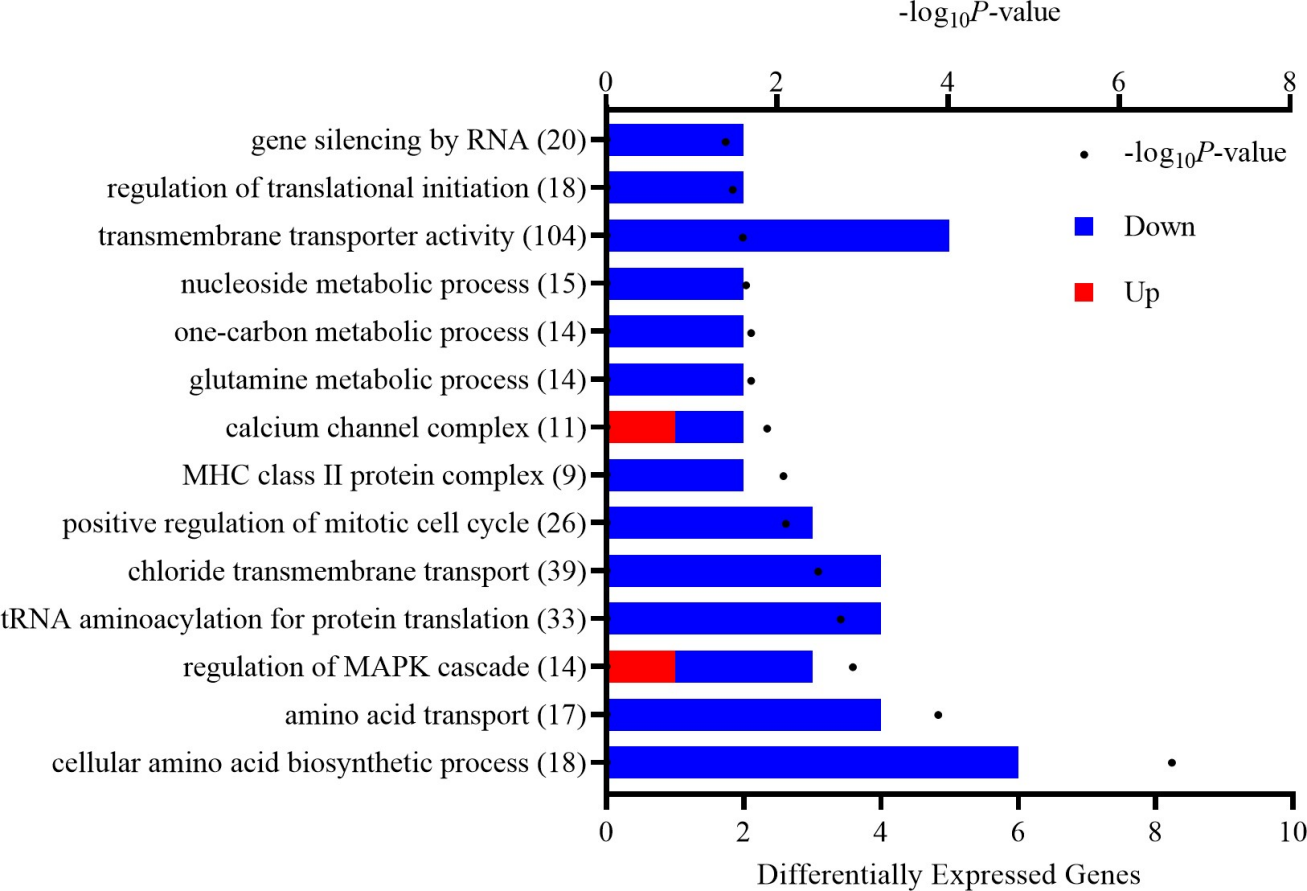
Significantly enriched gene ontology (GO) terms in the *Longissimus dorsi* of early-weaned *Bos indicus*-influenced calves for the effect of maternal supplementation with methionine hydroxy analog. The number of differentially expressed genes (*P* ≤ 0.05) is reported along the bottom x-axis and is depicted by the bars while the -log_10_*P*-value is reported along the top x-axis and is indicated by the dots. Blue bars indicate downregulated genes while red bars indicate upregulated genes. The number in parentheses is the total number of genes associated with each functional term.

Differentially expressed genes (*P* ≤ 0.05; 218 genes) were used to evaluate functional processes enriched in calves on the MOLMET vs. MOL treatment. Enrichment analysis revealed 8 significant KEGG pathways (Table 2) and 59 significant GO terms (Fig 2). Methionine supplementation influenced KEGG pathways associated with amino acid synthesis and metabolism (e.g., *biosynthesis of amino acids*, *glycine*, *serine*, *and threonine metabolism*, and *cysteine and methionine metabolism*) and the one-carbon cycle (e.g., *one-carbon pool by folate*). Significant GO terms were associated with protein synthesis (e.g., *regulation of translation initiation* and *tRNA aminoacylation for protein translation*) as well as transport across the cell membrane (e.g., *amino acid transport*, *transmembrane transporter activity* and *chloride transmembrane transporter*). Interestingly, a greater percentage of DEG involved in these functional processes were downregulated with methionine fortification.

**Table 2:** Significantly enriched Kyoto Encyclopedia of Genes and Genomes (KEGG) pathways in the *Longissimus dorsi* muscle of early-weaned *Bos indicus*-influenced calves for the effect of maternal supplementation with methionine hydroxy analog^1^.

		Differentially expressed genes^2^		
KEGG term	No. DEG	Upregulated	Downregulated	*P*-value
Biosynthesis of amino acids	8	-	*PYCR1*, *CBS*, *ASNS*, *PSPH*, *ALDOC*, *PSAT1*, *PHGDH*, *PRPS2*	<0.001
Glycine, serine and threonine metabolism	4	-	*CBS*, *PSPH*, *PSAT1*, *PHGDH*	0.001
One carbon pool by folate	3	-	*MTHFD2*, *ALDH1L2*, *MTHFD1L*	0.003
Cysteine and methionine metabolism	4	-	*CBS*, *PSAT1*, *MTAP*, *PHGDH*	0.005
Aminoacyl-tRNA biosynthesis	4	*-*	*CARS1*, *SARS1*, *TARS1*, *GARS1*	0.007
Carbon metabolism	5	*-*	*PSPH*, *ALDOC*, *PSAT1*, *PHGDH*, *PRPS2*	0.026
Steroid biosynthesis	2	*-*	*SOAT1*, *DHCR7*	0.031
Protein digestion and absorption	3	*DPP4*	*ATP1A1*, *SLC3A2*	0.046

^1^Treatments consisted of: no supplementation (**NOSUP**); supplementation of protein and energy using sugarcane molasses + urea (**MOL**; 7.2 kg of DM/heifer/wk; Westway Feed Products LLC, Clewiston, FL); or MOL fortified with 105 g/heifer/wk of methionine hydroxy analog (**MOLMET**; MFP, Novus International Inc., Romance, AR). Treatments were initiated on d 0 (57 ± 5 d prepartum) and continued until all heifers within a pasture had calved (17 ± 5 d postpartum).

^2^Threshold for differentially expressed genes was set at *P* ≤ 0.05

#### Effects of time (pre- vs. post-vaccination)

**Fig 3:**
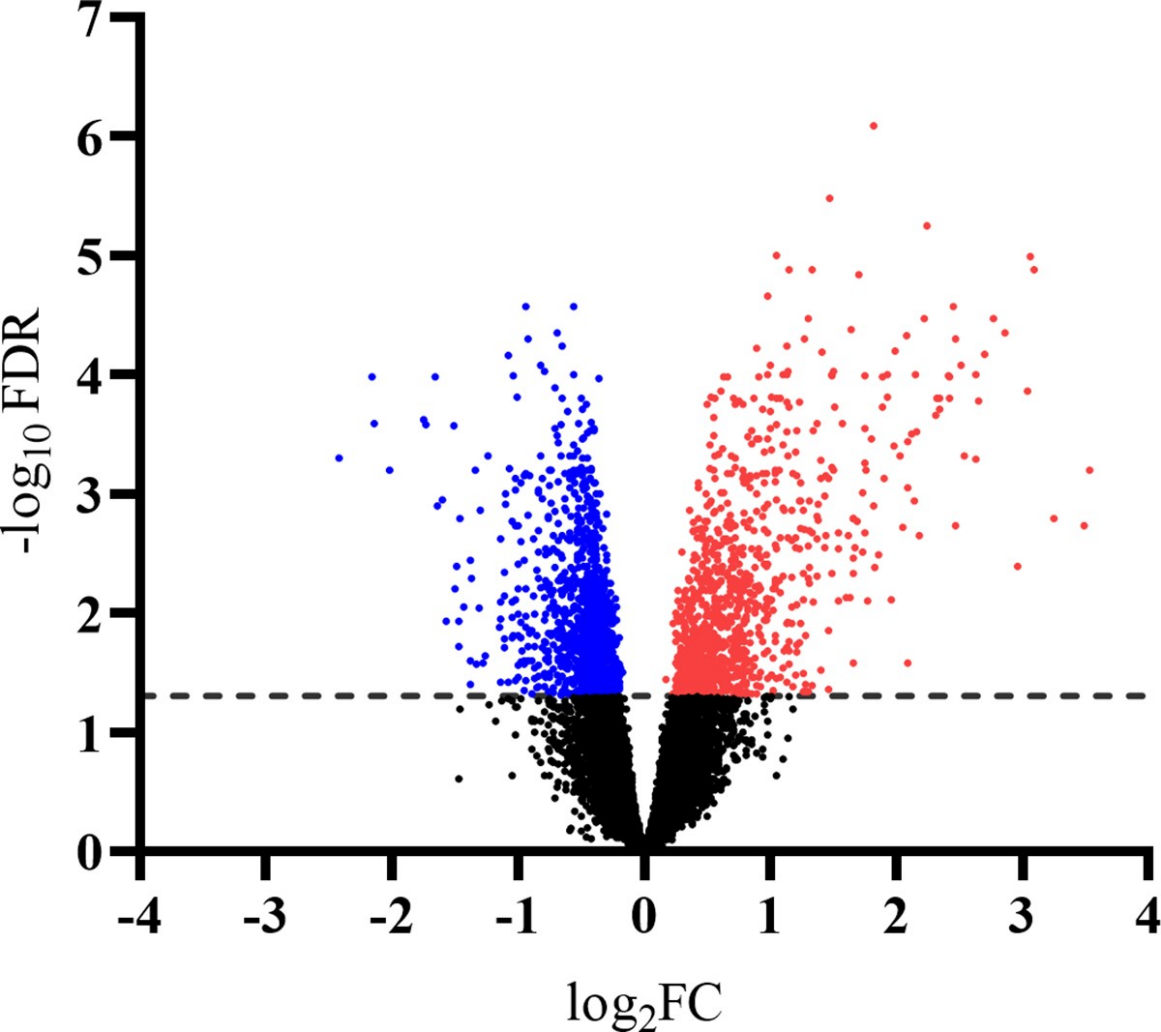
Volcano plot representing the differently expressed genes due to the main effect of time (pre- vs. post-vaccination). A total of 2,396 genes were differently expressed after vaccination (d 201 vs. d 154). The cutoff for DEG was considered at FDR ≤ 0.05, as indicated by the dash line. The y-axis is the -log_10_FDR while the x-axis represents the log_2_FC. Each dot represents a gene. Blue dots indicate downregulated genes, red dots indicate upregulated genes, and black dots indicate non-differentially expressed genes on d 201.

**Fig 4:**
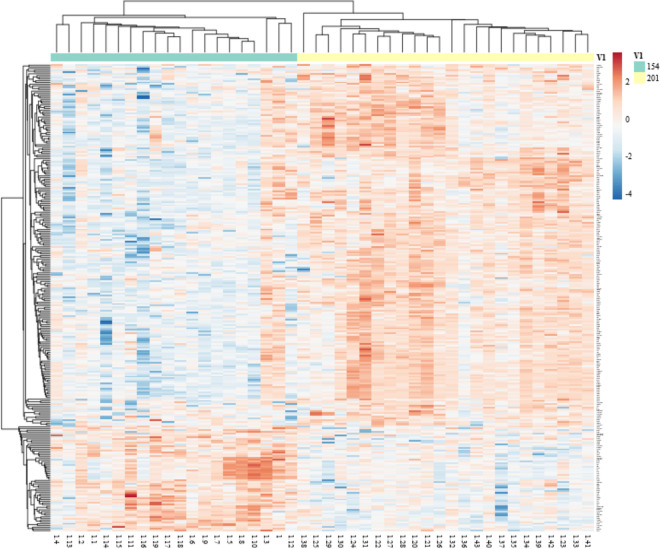
Heatmap of differently expressed genes with a fold change restriction [FDR ≤ 0.05 and a log_2_ fold change (log_2_FC) ≥ |1|] in the longissimus muscle of calves on d 154 and d 201. Each row represents a differentially expressed gene, and each column is an individual calf. Columns under the green bar represent samples collected on d 154 and columns under the yellow bar represent samples collected on d 201. Red indicates upregulated genes and blue represents downregulated genes. Rows and columns are clustered using correlation distance and average linkage.

Muscle transcriptome was highly impacted by the effect of time (pre- vs. post-vaccination) in *Bos indicus*-influenced calves. A total of 2,396 genes were differentially expressed (FDR ≤ 0.05) on d 201 (post-vaccination) compared to d 154 (pre-vaccination) as represented by the volcano plot in Fig 3. Out of the total DEG post-vaccination, approximately 1,336 genes were downregulated while 1,060 genes were upregulated. The heatmap depicted in Fig 4 represents the difference in gene expression from all calves sampled on d 154 and 201 for genes that had an FDR ≤ 0.05 and a log_2_FC ≥ |1|. When employing a log_2_FC ≥ |1| restriction, 270 genes are reported as differentially expressed on d 201 compared to d 154. Of the 270 genes, 61 were downregulated and 209 were upregulated (S2 Table). Several genes involved in both the innate and adaptive immune system were differentially expressed after vaccination. Downregulated genes involved in immune response included *IFI6*, *IFITM1*, *MST1*, *IGHG1*, and *CCRL2*, whereas genes *NFKB2*, *CCL2*, *IGDCC4* and *CD83* were upregulated. Additionally, two unclassified genes involved in the complement cascade, *LOC107131209* and *LOC781663*, were downregulated after vaccination. Genes encoding for transcription factors were upregulated including, members of the *FOS* family (e.g., *FOS*, *FOSB*, and *FOSL1*), *JUNB*, *IRF7*, and *EGR1*. Genes involved in the regulation of cell cycle division (e.g., *CCNA2*, *CCND1*, *CCND2*, and *CDC20*) as well as formation and organization of the extracellular matrix (e.g., *COL1A2*, *COL8A1*, *FBN3*, *FN1*, and *NPNT*) were upregulated after vaccination. Further, genes associated with energy metabolism were upregulated on d 201 such as *PFKFB3*, *INSIG1*, and *GCGR*.

**Fig 5:**
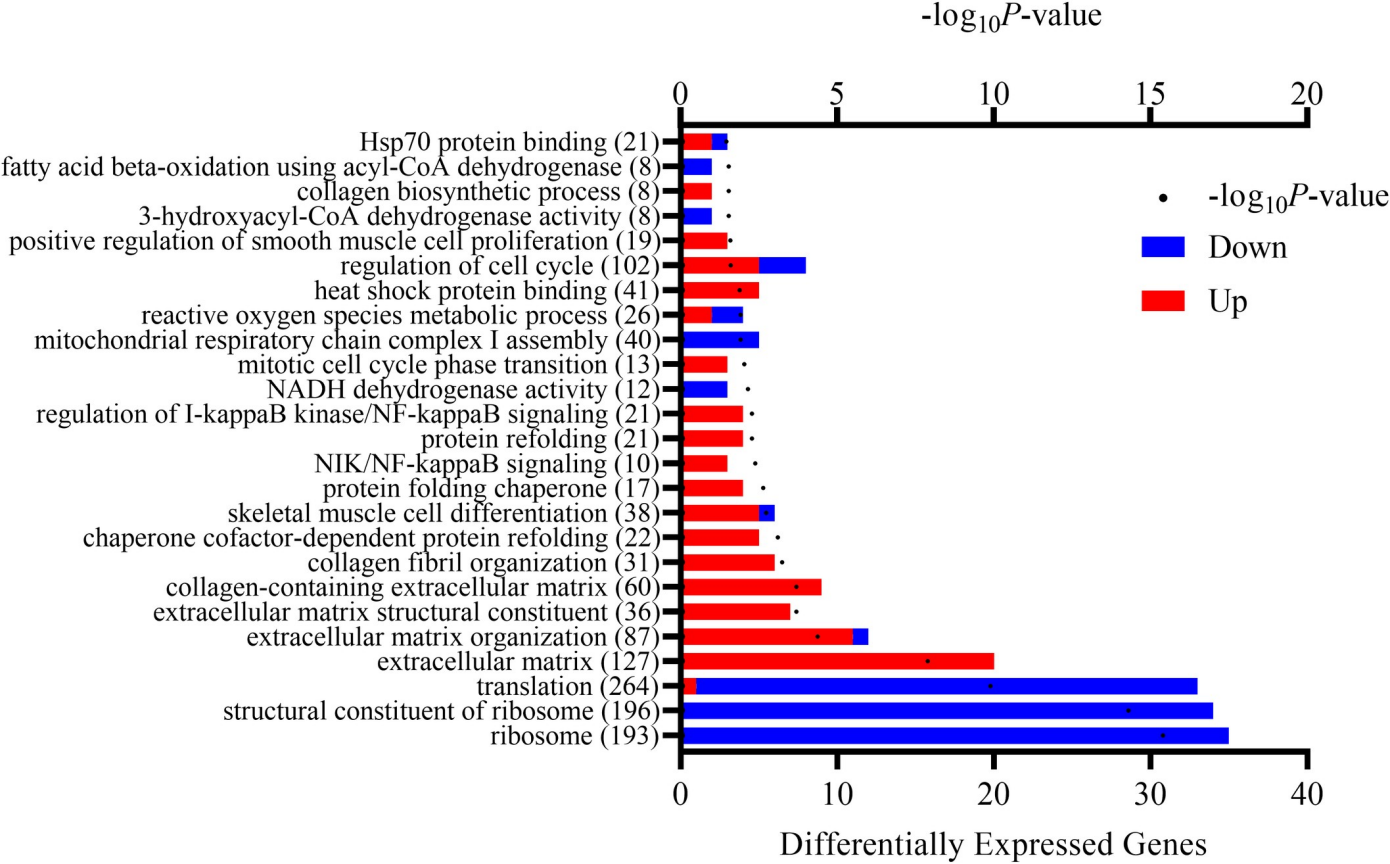
Significantly enriched gene ontology (GO) terms in the *Longissimus dorsi* muscle of early-weaned *Bos indicus*-influenced calves for the main effect of time (pre- vs. post-vaccination). The number of differentially expressed genes (*P* ≤ 0.0001) is reported along the bottom x-axis and depicted by bars while the -log_10_*P*-value is reported along the top x-axis is indicated by dots. Blue bars indicate downregulated genes while red bars indicate upregulated genes. The number in parentheses is the total number of genes associated with each functional term.

Differentially expressed genes (*P* ≤ 0.0001; 452 genes) based on the main effect of time (pre- and post- vaccination) generated 7 KEGG pathways (Table 3) and 180 GO terms (Fig 5). *Ribosome* was the most significant GO term and KEGG pathway identified by gene-set enrichment analysis and all genes associated with this pathway were downregulated after vaccination. Further, there was an overrepresentation of genes involved in pathways related to cell cycle. A greater percentage of genes associated with cell cycle pathways were upregulated on d 201 compared to d 154. There was a significant overrepresentation of genes associated with collagen formation and the extracellular matrix on d 201. Lastly, both GO and KEGG enrichment analysis reported functional processes associated with energy metabolism, including KEGG pathway *fatty acid degradation* and GO term *fatty acid beta-oxidation using acyl-CoA dehydrogenase*.

**Table 3:** Significantly enriched Kyoto Encyclopedia of Genes and Genomes (KEGG) pathways in the *Longissimus dorsi* muscle of early-weaned *Bos indicus*-influenced calves for the effect of time (pre- vs. post- vaccination)^1^.

		Differentially expressed genes^2^		
KEGG term	No. DEG	Upregulated	Downregulated	*P*-value
Ribosome	30	-	*MRPL23*, *RPS12*, *RPL21*, *MRPL10*, *RPL14*, *MRPL21*, *RPS3*, *RPL10*, *UBA52*, *RPS14*, *RPS2*, *RPSA*, *RPS19*, *RPS11*, *RPS8*, *RPL18*, *RPS10*, *RPL18A*, *RPS5*, *MRPL28*, *RPLP0*, *RPS20*, *RPL10A*, *RPS15*, *RPS16*, *RPL28*, *MRPS15*, *RPS25*, *LOC101902561*, *RPS13*	<0.0001
Valine, leucine and isoleucine degradation	7	-	*HADH*, *IVD*, *MCCC1*, *ALDH7A1*, *BCKDHB*, *MMUT*, *HSD17B10*	0.001
ECM-receptor interaction	7	*TNC*, *THBS1*, *NPNT*, *FN1*, *COL1A1*, *COL1A2*, *SDC4*	*-*	0.010
Neuroactive ligand-receptor interaction	8	*LPAR1*, *GRIA3*, *HRH2*	*GCGR*, *VIPR1*, *LPAR6*, *CHRND*, *PTH1R*	0.016
Complement and coagulation cascades	5	*SERPINE1*	*LOC107131209*, *MASP1*, *FGB*, *LOC781663*	0.019
Nitrogen metabolism	2	*CA3*	*CA14*	0.031
Fatty acid degradation	4	-	*HADH*, *ALDH7A1*, *ECI1*, *GCDH*	0.045

^1^Treatments consisted of: no supplementation (**NOSUP**); supplementation of protein and energy using sugarcane molasses + urea (**MOL**; 7.2 kg of DM/heifer/wk; Westway Feed Products LLC, Clewiston, FL); or MOL fortified with 105 g/heifer/wk of methionine hydroxy analog (**MOLMET**; MFP, Novus International Inc., Romance, AR). Treatments were initiated on d 0 (57 ± 5 d prepartum) and continued until all heifers within a pasture had calved (17 ± 5 d postpartum).

^2^Threshold for differentially expressed genes was set at *P* ≤ 0.0001

## Discussion

### Effects of maternal protein and energy supplementation

Overall, we acknowledge that there are limitations with the interpretation of our results considering that a less restrictive threshold was used to investigate the biological impact of maternal supplementation with energy and protein and maternal supplementation with methionine. However, the use of mixed models with orthogonal contrasts to investigate gene expression data is relatively novel. Protein and energy supplementation of heifers coincided with the last trimester of gestation when the fetus is growing at an accelerated rate [36]. Our research group has shown that providing additional protein and energy, in the form of a molasses + urea supplement, during the last trimester of gestation modulated the postnatal growth and physiology of beef calves born to primiparous and multiparous cows [22, 23]. Our results reveal genes in pathways relating to muscle differentiation, intracellular signaling, and epigenetic biology were modified with pre- and postpartum protein and energy supplementation in the *Longissimus dorsi* muscle of beef calves.

Pre- and postpartum supplementation of protein and energy led to an overrepresentation of downregulated genes in association with muscle development, such as *skeletal muscle cell differentiation*, *skeletal muscle cell development*, *muscle organ development* and *striated muscle cell differentiation*. Further observation into the functional processes associated with muscle development revealed that *MYF5*, a myogenic regulatory factor (**MRF**), was downregulated in calves born to heifers supplemented with molasses compared to calves born to heifers on the NOSUP treatment. Gene *MYF5*, along with other MRF, regulate the differentiation and proliferation of muscle satellite cells [37, 38]. In addition, several genes (e.g., *EGR1*, *SIX1*, *SIX4* and *NR4A1*) encoding for transcription factors involved in the regulation of MRF expression [39–43], were also downregulated with maternal supplementation of protein and energy. In support, Paradis et al. reported greater expression of genes *MYOG* and *MYOD1* in the *Longissimus dorsi* muscle of fetuses when cows were fed a low vs. high energy diet (85% vs. 140% of total metabolizable energy requirements, respectively) from mid- to late gestation, although no differences in phenotype were observed [8]. Further, expression of *MYOG* was increased in the longissimus muscle of fetuses when cows were offered 72% vs. 87% of their total energy requirements from d 85 to 180 of gestation [7]. Collectively, these studies suggest that improving the nutrient status of the dam during mid- to late gestation could negatively impact genes related to muscle development. However, more work is needed to evaluate how the duration and extent of maternal nutrient restriction or excess influences genes related to muscle development.

In the current study, calves born to heifers offered molasses supplementation had greater postweaning body weight gain compared to calves born to heifers on the NOSUP treatment [22]. Hence, it was unexpected to observe that genes associated with muscle differentiation and development were downregulated in the *Longissimus dorsi* muscle of calves born to heifers supplemented molasses vs. calves born to heifers on the NOSUP treatment. Longissimus muscle area and muscle fiber characteristics were not measured during this study; therefore, it is unknown how changes in gene expression influenced the actual longissimus muscle tissue mass. However, previous research reported that calves born to cows that received a protein supplement during the last trimester of gestation had greater weaning and carcass weights, but there was no difference in the longissimus muscle area at time of harvest [2, 4], suggesting that the greater body weight gain of calves born from supplemented cows may be explained by other mechanisms besides differences in muscle tissue growth. Further, genes related to myogenesis can respond differently to maternal nutrition based on muscle tissue type [8]. It is plausible that different muscle tissues responded differently to maternal supplementation of energy and protein, which could have led to an increase in calf post-weaning body weight.

Muscle, adipose, and connective tissue are all derived from mesenchymal stem cells and are considered competitive processes regulated by various signaling pathways [44, 45]. Pathway enrichment analysis revealed an overrepresentation of downregulated DEG involved in Wnt signaling, transforming growth factor (**TGF**)–β signaling and bone morphogenic protein (**BMP**) signaling in calves born to heifers that received energy and protein supplementation vs. NOSUP calves. The expression of each pathway has differing effects on muscle growth and development. In general, activation of the Wnt/β-catenin pathway enhances myogenesis and promotes the expression of MRF while inhibition of the Wnt/β-catenin pathway increases expression of adipogenic factors [46, 47]. On the other hand, activation of TGF-β signaling suppresses muscle growth and promotes the proliferation of fibroblasts [48]. The BMP are a subfamily of the TGF–β superfamily; however, BMP signaling promotes muscle fiber hypertrophy [49]. Previous work in both the bovine and ovine model has shown that the Wnt/β-catenin and TGF- β signaling pathways are susceptible to changes when modifications are made to the maternal diet during gestation. In fact, maternal overnutrition during gestation downregulates Wnt/β-catenin signaling and increases the expression of genes associated with adipogenesis and fibrogenesis in the fetus [50, 51]. Whereas, maternal obesity in pregnant ewes activated the TGF-β pathway and increased collagen formation in the semitendinosus muscle of fetuses [52]. In the present study, heifers were not considered obese prior to calving, however, this study suggests that even subtle increases in the nutrient status of heifers during gestation can have long-lasting effects on signaling pathways that influence muscle development. Moreover, all DEG related to WNT, TGF-β, and BMP signaling were downregulated, potentially indicating a downregulation in myogenesis and an increase in adipocyte differentiation. However, muscle phenotype (i.e., muscle mass and adipocyte population) was not investigated in the present study and further work is warranted to determine how genes modified in the current study affected muscle and adipose development.

It has been established that alterations to the mothers’ diet during the last trimester of gestation can cause alterations in gene expression [9, 53]. In the present study, there was a significant downregulation of DEG in the muscle tissue of calves born to heifers that received molasses compared to calves on the NOSUP treatment. Pathway enrichment analysis revealed a downregulation of DEG in functional terms related to gene transcription, such as *positive regulation of transcription*, *DNA- binding transcription factor activity*, and *RNA polymerase II proximal promoter sequence-specific DNA binding*. One potential mechanism to explain how maternal environment modulates gene expression is through epigenetic modifications, including DNA methylation, histone and chromatin modifications, and regulation of non-coding RNA [54]. Interestingly, we identified individual genes and pathways closely related to DNA methylation, chromatin remodeling, and histone methylation that were influenced by molasses supplementation.

Pathway enrichment analysis identified functional terms related to chromatin biology (*chromatin*, *chromatin binding*). Within the *chromatin binding* pathway there were several downregulated genes of interest, including: (1) *ANDP*, which interacts with members of the chromatin remodeling complex [55], (2) *PHF21A*, a gene involved in regulating histone methylation [56], and (3) *ASXL2*, which facilitates the binding of the Polycomb Repressive Complex 2 to the promoter region and subsequently represses gene expression [57]. Further, histone methylation (*histone methyltransferase activity* [*H3-K36 specific*] and *methylated histone binding*) was another process altered in calves born to heifers offered molasses supplementation vs. NOSUP. In particular, methylation of histone H3K36 plays a role in the activation of gene body transcription [58]. Genes *ASH1L* and *NSD1* were downregulated within the *histone methyltransferase activity* [*H3-K36 specific*] pathway and encode for methyltransferases specific for mono- and di-methylation of histone H3K36 [59]. While histone modifications were not measured in the present study, several changes in genes related to epigenetic biology provide evidence that maternal nutrition impacts genes involved in histone and chromatin modifications which can have long-term implications on offspring gene expression.

Further, methylation of DNA is largely dependent on the availability of methyl donors and is therefore influenced by maternal nutrition. Lan et al. reported differences in methylation in the fetus of ewes offered different isoenergetic diets during the last trimester of gestation [60]. They found that fetuses from ewes offered alfalfa haylage and dried distillers grains had increased methylation compared to fetuses from ewes offered corn, which they attributed to a greater amino acid intake [60]. Generally, when DNA methylation occurs in the promoter region it leads to the silencing of genes [61], which would explain the significant downregulation of genes observed with molasses supplementation. However, when we further investigate genes associated with pathways involved in gene transcription, we observed that *DNMT3a* was downregulated in calves born to heifers that received molasses supplementation vs. NOSUP calves. Gene *DNMT3a* encodes for DNA methyltransferase 3a, which is one enzyme responsible for de novo DNA methylation [62]. In the present study, DNA methylation was not measured, thus it is unknown how a reduction in *DNMT3a* affected methylation in the muscle tissue of calves.

### Effects of methionine fortification of supplements

The most significant pathways enriched in the muscle of calves born to heifers on the MOLMET treatment compared to calves born to heifers on the MOL treatment were composed of genes related to amino acid transport, synthesis, and metabolism. Within the functional term *amino acid transport*, two downregulated genes, *SLC7A5* and *SLC3A2*, encode for proteins that form a transmembrane complex responsible for the delivery of large neutral amino acids (namely, histidine, methionine, leucine, isoleucine, valine, phenylalanine, tyrosine, tryptophan, and glycine). In addition, *SLC7A1* was also downregulated in calves on the MOLMET treatment compared to the calves on the MOL treatment. Gene *SLC7A1* encodes for a transmembrane protein that mediates the transport of cationic amino acids (arginine, lysine, and histidine). In general, amino acid transporters facilitate the transport of amino acids, but they also act as intra- and extracellular amino acid sensors, thus playing an active role in the regulation of amino acid metabolism in the muscle cell [63]. For instance, expression of the SLC7A5 transporter in the skeletal muscle of rats influences the mammalian target of rapamycin (mTOR) signaling pathway [64], which is critical for the initiation of protein synthesis and muscle hypertrophy. Thus, the downregulation of genes involved in amino acid transport could have influenced downstream processes that require amino acids.

Synthesis and metabolism of amino acids were biological processes (e.g., *biosynthesis of amino acids*, *glycine*, *serine*, *and threonine metabolism*, *cysteine and methionine metabolism*, and *glutamine metabolic process*) modified in the muscle tissue of calves born to heifers on the MOLMET treatment compared to calves born to heifers on the MOL treatment. Among these processes, there were four genes, *PSPH*, *PSAT1*, *PHGDH*, and *CBS*, that were consistently downregulated in calves on the MOLMET treatment vs. the MOL treatment. Genes *PSPH*, *PSAT1*, and *PHGDH* encode enzymes responsible for the production of the nonessential amino acid serine from 3-P-glycerate [65]. While *CBS* encodes for the enzyme, cystathionine β-synthase, which converts homocysteine, an intermediate in the methionine cycle, and serine to cystathionine in the first step of the transsulfuration pathway [66]. The downregulation of *CBS* in the current study could indicate a reduction in the transsulfuration pathway, which would suggest an increase in the remethylation of methionine [66]. In contrast, maternal supply of methionine (offered at 0.09% of the diet DM) during the last trimester of gestation increased expression of *CBS* along with metabolites found in the transsulfuration pathway in the liver of dairy calves [18]. It is possible that breed impacted the differences in gene expression observed between the two studies. However, discrepancies between the two studies could also suggest that maternal supplementation with methionine during the last trimester of gestation affects the metabolism of amino acids differently based on tissue type.

This study revealed several functional terms related to protein synthesis, such as *regulation of translational initiation* and *tRNA aminoacylation for protein translation*. Of further importance, all DEG corresponding to protein synthesis were downregulated in calves on the MOLMET treatment compared to calves on the MOL treatment. Body weight gain is positively correlated to protein deposition, which is achieved when protein synthesis occurs at a greater rate than protein degradation [67]. Therefore, a reduction in genes related to protein synthesis in calves on the MOLMET vs. MOL could have impacted postnatal growth. Utilizing calves from the current study, our research group reported that post-weaning body weight gain did not differ between calves born to heifers that received or not methionine supplementation [22]. In support, maternal supplementation of methionine during the last trimester of gestation in multiparous beef cows did not affect calf body weight gain [21, 23]. It remains possible that a reduction in the expression of genes related to protein synthesis in the muscle of MOLMET vs. MOL calves could partially explain why maternal supplementation with methionine did not further enhance postnatal body weight gain. However, additional mechanisms were likely involved because, as we discussed previously, maternal gestational supplementation of protein and energy downregulated genes associated with myogenesis, but simultaneously enhanced calf postnatal growth performance compared to calves born from non-supplemented heifers.

The one carbon cycle is regulated through the availability of methyl donors in the diet, such as methionine. Hence, it was not surprising that calves born to heifers on the MOLMET treatment had an overrepresentation of DEG involved in one-carbon metabolism. Interestingly, all genes (*MTHFD2*, *ALDH1L2*, and *MTHFD1L*) were downregulated in calves on the MOLMET treatment and encode for enzymes in the mitochondrial folate pathway [68–70]. The mitochondrial folate pathway produces formate, which is used by the cytosolic folate pathway in the regeneration of 10-formyl tetrahydrofolate [71]. The folate cycle is required for several metabolic processes in the body including the synthesis of purines and pyrimidines and the regeneration of methionine. Interestingly, the functional term *nucleoside metabolic process* was enriched in calves on the MOLMET vs. MOL treatments and all DEG were downregulated.

### Effects of time (pre- vs. post-vaccination)

Genes and pathways connected to energy metabolism were influenced by vaccination. There was an overrepresentation of downregulated genes related to processes involved in fatty acid metabolism, such as *fatty acid degradation* and *fatty acid beta-oxidation using acyl-CoA biosynthetic process*. Individual genes regulating glucose homeostasis (e.g., *PFKFB3* and *GCGR*) were also upregulated on d 201. *PFKFB3* encodes for a gene that stimulates the synthesis of fructose-6,2-bisphosphosphate and promotes glycolysis [72]. *PFKFB3* has further been identified as a target for PPARy, a protein responsible for the initiation of adipocyte differentiation [73]. Additionally, *INSIG1*, a gene involved in energy metabolism, was upregulated on d 201 vs. d 154. Gene *INSIG1* is a target of PPARy and is associated with adipogenesis in the muscle tissue of early weaned calves [74].

In the present study, there was an upregulation of genes related to the extracellular matrix, collagen formation, and cell cycle regulation in the muscle of calves after vaccination. Guo et al. identified gene-sets involved in the cell cycle process and extracellular matrix to be co-expressed in muscle tissue and positively correlated to average daily gain in Brahman steers [75]. Using known cell marker types, authors proposed that the functional processes of cell cycle and extracellular matrix were derived predominately from fibroblasts during postnatal muscle development [75]. Collectively our results suggest that there was an increase in genes related to adipogenesis and fibrogenesis in the *longissimus dorsi* muscle after vaccination. It is important to note that muscle biopsies were collected from the same area of the *longissimus dorsi* on d 154 and 201; thus, the upregulation of genes related to fibrogenesis could be partially due to changes in the muscle tissue following the first muscle biopsy.

Ribosome structure and translation were among the top functional processes enriched on d 201 vs. d 154. Within those pathways, most DEG were downregulated, possibly indicating a reduction in protein synthesis in muscle tissue on d 201. Calves did exhibit positive body weight gain from d 154 to d 201, however, average daily gain of calves observed in the present study was reduced compared to earlier studies conducted by our research group where calves were early-weaned and offered a high-concentrate diet [76, 77]. Unlike the present study, calves from earlier studies were not subjected to a vaccination challenge. Vaccination stimulates an acute phase protein response which is a component of the innate immune system [78]. Thus, it is plausible that the reduction in genes related to protein synthesis in the muscle tissue of calves on d 201 is due to the repartitioning of nutrients towards the immune system and away from growth [25]. This is further supported by the fact that time of sample collection had a significant influence on the expression of individual genes related to both the innate and adaptive immune response. Further, there as an overrepresentation on d 201 of upregulated genes related to the NF-κB signaling pathway, which is also a component of the inflammatory response.

## Conclusions

In conclusion, this study revealed that maternal pre- and postpartum supplementation of energy and protein, with or without methionine fortification, altered the muscle transcriptome of *Bos indicus*-influenced beef calves. Maternal supplementation of energy and protein impacted genes involved in biological processes related to muscle development, intracellular signaling pathways, and epigenetic modifications. Maternal supplementation with methionine downregulated gene-sets involved in the transport, synthesis, and metabolism of amino acids as well as the one-carbon cycle. Vaccination had a significant impact on the longissimus muscle transcriptome of beef calves and influenced genes associated with ribosome structure, translation, extracellular matrix, collagen formation, and immune function. This study revealed genes and pathways in the muscle tissue of offspring influenced by maternal nutrition and vaccination. Further research is needed to understand how these modifications might translate into phenotypic changes in the offspring.

## Supporting information

S1 Table Mapping summary. (XLSX)

S2 Table List of differentially expressed genes. Significant genes for the effect of maternal supplementation with protein and energy vs. NOSUP (*P* ≤ 0.01; Tab 1), maternal supplementation with methionine hydroxy analog vs. MOL (*P* ≤ 0.01; Tab 2) and time (d 201 vs. d 154; FDR ≤ 0.05; Tab 3) in the *longissimus dorsi* muscle of early-weaned *Bos indicus*-influenced calves. An additional table is available that lists differentially expressed genes on d 201 vs. d 154 when a restriction on fold change is employed (Tab 4; FDR ≤ 0.05; log_2_ fold change (log_2_FC) ≥ |1|).
(XLSX)

S3 Table List of Gene Ontology (GO) terms significantly enriched with differentially expressed genes. Significant GO terms with the up and downregulated genes enriched in each term for the effect of maternal supplementation with protein and energy (*P* ≤ 0.025), maternal supplementation with methionine (*P* ≤ 0.05) and time (FDR ≤ 0.0001) in the *longissimus dorsi* muscle of early-weaned *Bos indicus*-influenced calves.
(XLSX)

## References

[1] NASEM (National Acadamies od Sciences, Engineering, and Medicine). Nutrient Requirements of beef cattle. 2016. 8th ed. Animal Nutrition Series. Washington (DC): The National Academies Press.

[2] Stalker LA, Ciminski LA, Adams DC, Klopfenstein TJ, Clark RT. Effects of weaning date and prepartum protein supplementation on cow performance and calf growth. Rangeland Ecol Manage. 2007;60:578–587. doi: 10.2111/06-082R1.1

[3] Stalker LA, Adams DC, Klopfenstein TJ, Feuz DM, Funston RN. Effects of pre-and postpartum nutrition on reproduction in spring calving cows and calf feedlot performance. J Anim Sci. 2006;84:2582–2589. doi: 10.2527/jas.2005-640 16908664 PMC7109832

[4] Larson DM, Martin JL, Adams DC, Funston RN. Winter grazing system and supplementation during late gestation influence performance of beef cows and steer progeny. J Anim Sci. 2009;87:1147–1155. doi: 10.2527/jas.2008-1323 18997078 PMC7110207

[5] Wegner J, Albrecht E, Fiedler I, Teuscher F, Papstein HJ, Ender K. Growth- and breed-related changes of muscle fiber characteristics in cattle. J Anim Sci. 2000;78:1485–1496. doi: 10.2527/2000.7861485x 10875630

[6] Du M, Tong J, Zhao J, Underwood KR, Zhu M, Ford SP, et al. Fetal programming of skeletal muscle development in ruminant animals. J Anim Sci. 2010;88(E. Suppl.):E51–E60. doi: 10.2527/jas.2009-2311 19717774

[7] Jennings TD, Gonda MG, Underwood KR, Wertz-Lutz AE, Blair AD. The influence of maternal nutrition on expression of genes responsible for adipogenesis and myogenesis in the bovine fetus. Animal. 2016;10:1697–1705. doi: 10.1017/S1751731116000665 27121146

[8] Paradis F, Wood KM, Swanson KC, Miller SP, McBride BW, Fitzsimmons C. Maternal nutrient restriction in mid-to-late gestation influences fetal mRNA expression in muscle tissues in beef cattle. BMC Genomics. 2017;18:632. doi: 10.1186/s12864-017-4051-5 28821223 PMC5562975

[9] Sanglard LP, Nascimento M, Moriel P, Sommer J, Ashwell M, Poore MH, et al. Impact of energy restriction during late gestation on the muscle and blood transcriptome of beef calves after preconditioning. BMC Genomics. 2018;19:702. doi: 10.1186/s12864-018-5089-8 30253751 PMC6156876

[10] Waterman RC, Löest CA, Bryant WD, Petersen MK. Supplemental methionine and urea for gestating beef cows consuming low quality forage diets. J Anim Sci. 2007;85:731–736. doi: 10.2527/jas.2006-425 17060412

[11] Waterman RC, Ujazdowski VL, Petersen MK. Effects of rumen-protected methionine on plasma amino acid concentrations during a period of weight loss for late gestating beef heifers. Amino Acids. 2012;43:2165–77. doi: 10.1007/s00726-012-1301-3 22555648

[12] Bach A, Aris A, Guasch I. Consequences of supplying methyl donors during pregnancy on the methylome of the offspring from lactating and non-lactating dairy cattle. PLoS ONE. 2017;12:e0189581. doi: 10.1371/journal.pone.0189581 29228040 PMC5724855

[13] Liu L, Amorín R, Moriel P, Dilorenzo N, Lancaster PA, Peñagaricano F. Differential network analysis of bovine muscle reveals changes in gene coexpression patterns in response to changes in maternal nutrition. BMC Genomics. 2020;21:684. doi: 10.1186/s12864-020-07068-x 33008289 PMC7531131

[14] Hoffman DR, Marion DW, Cornatzer WE, Duerre JA. S-adenosylmethionine and S-adenosylhomocysteine metabolism in isolated rat liver. Effects of L-methionine, L-homocysteine, and adenosine. J Biol Chem. 1980;255:10822–10827. doi: 10.1016/s0021-9258(19)70381-0 7430157

[15] Mato JM, Alvarez L, Ortiz P, Pajares MA. S-Adenosylmethionine synthesis: Molecular mechanisms and clinical implications. 1997;73:265–280. doi: 10.1016/s0163-7258(96)00197-0. 9175157

[16] Mentch SJ, Mehrmohamadi M, Huang L, Liu X, Gupta D, Mattocks D, et al. Histone methylation dynamics and gene regulation occur through the sensing of one-carbon metabolism. Cell Metab. 2015;22:861–873. doi: 10.1016/j.cmet.2015.08.024 26411344 PMC4635069

[17] Jacometo CB, Zhou Z, Luchini D, Corrêa MN, Loor JJ. Maternal supplementation with rumen-protected methionine increases prepartal plasma methionine concentration and alters hepatic mRNA abundance of 1-carbon, methionine, and transsulfuration pathways in neonatal Holstein calves. J Dairy Sci. 2017;100:3209–3219. doi: 10.3168/jds.2016-11656 28161170

[18] Alharthi AS, Coleman DN, Liang Y, Batistel F, Elolimy AA, Yambao RC, et al. Hepatic 1-carbon metabolism enzyme activity, intermediate metabolites and growth in neonatal Holstein dairy calves are altered by maternal supply of methionine during late pregnancy. J Dairy Sci. 2019;102:10291–10303. doi: 10.3168/jds.2019-16562 31477291

[19] Batistel F, Alharthi AS, Yambao RRC, Elolimy AA, Pan YX, Parys C, et al. Methionine supply during late-gestation triggers offspring sex-specific divergent changes in metabolic and epigenetic signatures in bovine placenta. J Nutr. 2019;149:6–17. doi: 10.1093/jn/nxy240 30608595

[20] Alharthi AS, Batistel F, Abdelmegeid MK, Lascano G, Parys C, Helmbrecht A, et al. Maternal supply of methionine during late-pregnancy enhances rate of Holstein calf development in utero and postnatal growth to a greater extent than colostrum source. J Anim Sci Biotechnol. 2018;9:83. doi: 10.1186/s40104-018-0298-1 30498570 PMC6251175

[21] Clements AR, Ireland FA, Freitas T, Tucker H, Shike DW. Effects of supplementing methionine hydroxy analog on beef cow performance, milk production, reproduction, and preweaning calf performance. J Anim Sci. 2017;95:5597–5605. doi: 10.2527/jas2017.1828 29293801 PMC6292334

[22] Moriel P, Vedovatto M, Palmer EA, Oliveira RA, Silva HM, Ranches J, et al. Maternal supplementation of energy and protein, but not methionine hydroxy analogue, enhanced postnatal growth and response to vaccination in Bos indicus-influenced beef offspring. J Anim Sci. 2020;98. doi: 10.1093/jas/skaa123 32309862 PMC7228674

[23] Palmer EA, Vedovatto M, Oliveira RA, Gouvea V, Silva HM, Vendramini JMB, et al. Maternal supplement type and methionine hydroxy analogue fortification effects on performance of BOS indicus-influenced beef cows and their offspring. Livest Sci. 2020;240:104176. doi: 10.1016/j.livsci.2020.104176

[24] Silva GM, Chalk CD, Ranches J, Schulmeister TM, Henry DD, DiLorenzo N, et al. Effect of rumen-protected methionine supplementation to beef cows during the periconception period on performance of cows, calves, and subsequent offspring. Animal. 2021;15:100055. doi: 10.1016/j.animal.2020.100055 33516019

[25] Elsasser TH, Caperna TJ, Li CJ, Kahl S, Sartin JL. Critical control points in the impact of the proinflammatory immune response on growth and metabolism. J Anim Sci. 2008;86(E. Suppl.):E105–E125. doi: 10.2527/jas.2007-0634 18344314

[26] Coleman DN, Lopreiato V, Alharthi A, Loor JJ. Amino acids and the regulation of oxidative stress and immune function in dairy cattle. J Anim Sci. 2020;98:S175–93. doi: 10.1093/jas/skaa138 32810243 PMC7433927

[27] Alharthi AS, Lopreiato V, Dai H, Bucktrout R, Abdelmegeid M, Batistel F, et al. Short communication: Supply of methionine during late pregnancy enhances whole-blood innate immune response of Holstein calves partly through changes in mRNA abundance in polymorphonuclear leukocytes. J Dairy Sci. 2019;102:10599–605. doi: 10.3168/jds.2018-15676 31447163

[28] Artioli LFA, Moriel P, Poore MH, Marques RS, Cooke RF. Decreasing the frequency of energy supplementation from daily to three times weekly impairs growth and humoral immune response of preconditioning beef steers. J Anim Sci. 2015;93:5430–5441. doi: 10.2527/jas.2015-9457 26641062

[29] Moriel P, Piccolo MB, Artioli LFA, Marques RS, Poore MH, Cooke RF. Short-term energy restriction during late gestation of beef cows decreases postweaning calf humoral immune response to vaccination. J Anim Sci. 2016;94:2542–2552. doi: 10.2527/jas.2016-0426 27285930

[30] Silva GM, Poore MH, Ranches J, Moriel P. Effects of timing of vaccination relative to weaning and post-weaning frequency of energy supplementation on growth and immunity of beef calves. J Anim Sci. 2018;96:318–330. doi: 10.1093/jas/skx021 29378006 PMC6140980

[31] Kim D, Langmead B, Salzberg SL. HISAT: A fast spliced aligner with low memory requirements. Nat Methods. 2015;12:357–360. doi: 10.1038/nmeth.3317 25751142 PMC4655817

[32] Anders S, Pyl PT, Huber W. HTSeq-A Python framework to work with high-throughput sequencing data. Bioinformatics. 2015;31:166–169. doi: 10.1093/bioinformatics/btu638 25260700 PMC4287950

[33] Robinson MD, McCarthy DJ, Smyth GK. edgeR: A Bioconductor package for differential expression analysis of digital gene expression data. Bioinformatics. 2009;26:139–140. doi: 10.1093/bioinformatics/btp616 19910308 PMC2796818

[34] Benjamini Y, Hochberg Y. Controlling the False Discovery Rate: A practical and powerful approach to multiple testing. J R Statist Soc. 1995;57:289–300. doi: 10.1111/j.2517-6161.1995.tb02031.x

[35] Metsalu T, Vilo J. ClustVis: A web tool for visualizing clustering of multivariate data using Principal Component Analysis and heatmap. Nucleic Acids Res. 2015;43:W566–W570. doi: 10.1093/nar/gkv468 25969447 PMC4489295

[36] Ferrell CL, Garrett WN, Hinman N. Growth, development and composition of the udder and gravid uterus of beef heifers during pregnancy. J Anim Sci. 1976;42:1477–1489. doi: 10.2527/jas1976.4261477x 931823

[37] Muroya S, Nakajima I, Chikuni K. Sequential expression of myogenic regulatory factors in bovine skeletal muscle and the satellite cell culture. Anim Sci J. 2002;73:375–381. doi: 10.1046/j.1344-3941.2002.00052.x

[38] Cornelison DDW, Wold BJ. Single-cell analysis of regulatory gene expression in quiescent and activated mouse skeletal muscle satellite cells. Dev Biol. 1997;191:270–283. doi: 10.1006/dbio.1997.8721 9398440

[39] Grifone R, Demignon J, Houbron C, Souil E, Niro C, Seller MJ, et al. Six1 and Six4 homeoproteins are required for Pax3 and Mrf expression during myogenesis in the mouse embryo. Development. 2005;132:2235–2249. doi: 10.1242/dev.01773 15788460

[40] Wei D, Ma X, Zhang S, Hong J, Gui L, Mei C, et al. Characterization of the promoter region of the bovine SIX1 gene: Roles of MyoD, PAX7, CREB and MyoG. Sci Rep. 2017;7:12599. doi: 10.1038/s41598-017-12787-5 28974698 PMC5626756

[41] Zhang W, Tong H, Zhang Z, Shao S, Liu D, Li S, et al. Transcription factor EGR1 promotes differentiation of bovine skeletal muscle satellite cells by regulating MyoG gene expression. J Cell Physiol. 2018;233:350–362. doi: 10.1002/jcp.25883 28256014

[42] Tontonoz P, Cortez-Toledo O, Wroblewski K, Hong C, Lim L, Carranza R, et al. The Orphan Nuclear Receptor Nur77 Is a Determinant of Myofiber Size and Muscle Mass in Mice. Mol Cell Biol. 2015;35:1125–1138. doi: 10.1128/MCB.00715-14 25605333 PMC4355536

[43] Cortez-Toledo O, Schnair C, Sangngern P, Metzger D, Chao LC. Nur77 deletion impairs muscle growth during developmental myogenesis and muscle regeneration in mice. PLoS ONE. 2017;12:e0171268. doi: 10.1371/journal.pone.0171268 28170423 PMC5295706

[44] Du M, Zhao JX, Yan X, Huang Y, Nicodemus LV, Yue W, et al. Fetal muscle development, mesenchymal multipotent cell differentiation, and associated signaling pathways. J Anim Sci. 2011;89:583–590. doi: 10.2527/jas.2010-3386 20852073 PMC4100697

[45] Yan X, Zhu M, Dodson MV, Du M. Developmental programming of fetal skeletal muscle and adipose tissue development. J Genomics. 2013;1:29–38. doi: 10.7150/jgen.3930 25031653 PMC4091428

[46] Ridgeway AG, Petropoulos H, Wilton S, Skerjanc IS. Wnt signaling regulates the function of MyoD and myogenin. J Biol Chem. 2000;275:32398–32405. doi: 10.1074/jbc.M004349200 10915791

[47] Ross SE, Hemati N, Longo KA, Bennett CN, Lucas PC, Erickson RL, et al. Inhibition of adipogenesis by Wnt signaling. Science. 2000;289:950–953. doi: 10.1126/science.289.5481.950 10937998

[48] Kollias HD, McDermott JC. Transforming growth factor-β and myostatin signaling in skeletal muscle. J Appl Physiol. 2008;104:579–87. doi: 10.1152/japplphysiol.01091.2007 18032576

[49] Winbanks CE, Chen JL, Qian H, Liu Y, Bernardo BC, Beyer C, et al. The bone morphogenetic protein axis is a positive regulator of skeletal muscle mass. J Cell Biol. 2013;203:345–357. doi: 10.1083/jcb.201211134 24145169 PMC3812980

[50] Tong JF, Yan X, Zhu MJ, Ford SP, Nathanielsz PW, Du M. Maternal obesity downregulates myogenesis and β-catenin signaling in fetal skeletal muscle. Am J Physiol Endocrinol Metab. 2009;296(4):E917–E924. doi: 10.1152/ajpendo.90924.2008 19176350 PMC2670630

[51] Duarte MS, Gionbelli MP, Paulino PVR, Serão NVL, Nascimento CS, Botelho ME, et al. Maternal overnutrition enhances mRNA expression of adipogenic markers and collagen deposition in skeletal muscle of beef cattle fetuses. J Anim Sci. 2014;92:3846–3854. doi: 10.2527/jas.2014-7568 25006073

[52] Huang Y, Yan X, Zhu MJ, McCormick RJ, Ford SP, Nathanielsz PW, et al. Enhanced transforming growth factor-β signaling and fibrogenesis in ovine fetal skeletal muscle of obese dams at late gestation. Am J Physiol Endocrinol Metab. 2010;298:E1254–E1260. doi: 10.1152/ajpendo.00015.2010 20371734 PMC2886526

[53] Peñagaricano F, Wang X, Rosa GJ, Radunz AE, Khatib H. Maternal nutrition induces gene expression changes in fetal muscle and adipose tissues in sheep. BMC Genomics. 2014;15:1034. doi: 10.1186/1471-2164-15-1034 25429728 PMC4301459

[54] Sinclair KD, Rutherford KMD, Wallace JM, Brameld JM, Stöger R, Alberio R, et al. Epigenetics and developmental programming of welfare and production traits in farm animals. Reprod Fertil Dev. 2016;28:1443–1478. doi: 10.1071/RD16102 27439952

[55] Mandel S, Gozes I. Activity-dependent neuroprotective protein constitutes a novel element in the SWI/SNF chromatin remodeling complex. J Biol Chem. 2007;282:34448–34456. doi: 10.1074/jbc.M704756200 17878164

[56] Lan F, Collins RE, Cegli RD, Alpatov R, Horton JR, Shi X, et al. Recognition of unmethylated histone H3 lysine 4 links BHC80 to LSD1-mediated gene repression. Nature. 2007;448:718–722. doi: 10.1038/nature06034 17687328 PMC2702779

[57] Lai H, Wang QT. Additional Sex Combs-Like 2 is required for Polycomb Repressive Complex 2 binding at select targets. PLoS ONE. 2013;8:e73983. doi: 10.1371/journal.pone.0073983 24040135 PMC3767597

[58] Edmunds JW, Mahadevan LC, Clayton AL. Dynamic histone H3 methylation during gene induction: HYPB/Setd2 mediates all H3K36 trimethylation. EMBO J. 2008;27:406–420. doi: 10.1038/sj.emboj.7601967 18157086 PMC2168397

[59] Wagner EJ, Carpenter PB. Understanding the language of Lys36 methylation at histone H3. Nat Rev Mol Cell Biol. 2012;13:115–126. doi: 10.1038/nrm3274 22266761 PMC3969746

[60] Lan X, Cretney EC, Kropp J, Khateeb K, Berg MA, Peñagaricano F, et al. Maternal diet during pregnancy induces gene expression and DNA methylation changes in fetal tissues in sheep. Front Genet. 2013;4:49. doi: 10.3389/fgene.2013.00049 23577020 PMC3617393

[61] Curradi M, Izzo A, Badaracco G, Landsberger N. Molecular mechanisms of gene silencing mediated by DNA methylation. Mol Cell Biol. 2002;22:3157–3173. doi: 10.1128/MCB.22.9.3157-3173.2002 11940673 PMC133775

[62] Okano M, Bell DW, Haber DA, Li E. DNA methyltransferases Dnmt3a and Dnmt3b are essential for de novo methylation and mammalian development. Cell. 1999;99:247–257. doi: 10.1016/s0092-8674(00)81656-6 10555141

[63] Dickinson JM, Rasmussen BB. Amino Acid Transporters in the Regulation of Human Skeletal Muscle Protein Metabolism. Curr Opin Clin Nutr Metab Care. 2013;16:638–644. doi: 10.1097/MCO.0b013e3283653ec5 24100668 PMC4164966

[64] Poncet N, Mitchell FE, Ibrahim AFM, McGuire VA, English G, Arthur JSC, et al. The catalytic subunit of the system L1 amino acid transporter (Slc7a5) facilitates nutrient signalling in mouse skeletal muscle. PLoS ONE. 2014;9:e89547. doi: 10.1371/journal.pone.0089547 24586861 PMC3935884

[65] Snell K, Fell DA. Metabolic control analysis of mammalian serine metabolism. Adv Enzyme Regul. 1990;30:13–32. doi: 10.1016/0065-2571(90)90006-n 2119548

[66] Jhee K, Kruger WD. The role of cystathionine β-synthase in homocysteine metabolism. Antioxid Redox Signal. 2005;7:813–822. doi: 10.1089/ars.2005.7.813 15890029

[67] Castro Bulle FCP, Paulino PV, Sanches AC, Sainz RD. Growth, carcass quality, and protein and energy metabolism in beef cattle with different growth potentials and residual feed intakes. J Anim Sci. 2007;85:928–36. doi: 10.2527/jas.2006-373 17178805

[68] Krupenko NI, Dubard ME, Strickland KC, Moxley KM, Oleinik NV, Krupenko SA. ALDH1L2 is the mitochondrial homolog of 10-formyltetrahydrofolate dehydrogenase. J Biol Chem. 2010;285:23056–23063. doi: 10.1074/jbc.M110.128843 20498374 PMC2906299

[69] Nilsson R, Jain M, Madhusudhan N, Sheppard NG, Strittmatter L, Kampf C, et al. Metabolic enzyme expression highlights a key role for MTHFD2 and the mitochondrial folate pathway in cancer. Nat Commun. 2014;5:3128. doi: 10.1038/ncomms4128 24451681 PMC4106362

[70] Bryant JD, Sweeney SR, Sentandreu E, Shin M, Ipas H, Xhemalce B, et al. Deletion of the neural tube defect-associated gene Mthfd1l disrupts one-carbon and central energy metabolism in mouse embryos. J Biol Chem. 2018;293:5821–5833. doi: 10.1074/jbc.RA118.002180 29483189 PMC5912453

[71] Ducker GS, Chen L, Morscher RJ, Ghergurovich JM, Esposito M, Teng X, et al. Reversal of Cytosolic One-Carbon Flux Compensates for Loss of the Mitochondrial Folate Pathway. Cell Metab. 2016;23:1140–1153. doi: 10.1016/j.cmet.2016.04.016 27211901 PMC4909566

[72] Atsumi T, Nishio T, Niwa H, Takeuchi J, Bando H, Shimizu C, et al. Expression of inducible 6-Phosphofructo-2-Kinase/Fructose-2,6-Bisphosphatase/PFKFB3 isoforms in adipocytes and their potential role in glycolytic regulation. 2005;54:3349–3357. doi: 10.2337/diabetes.54.12.3349 16306349

[73] Huo Y, Guo X, Xu K, Zhang J, Li H, Zhang W, et al. Involvement of inducible 6-phosphofructo-2-kinase in the anti-diabetic effect of PPARγ activation in mice. J Biol Chem. 2010;285:23711–23720. doi: 10.1074/jbc.M110.123174 20498376 PMC2911274

[74] Graugnard DE, Piantoni P, Bionaz M, Berger LL, Faulkner DB, Loor JJ. Adipogenic and energy metabolism gene networks in longissimus lumborum during rapid post-weaning growth in Angus and Angus × Simmental cattle fed high-starch or low-starch diets. BMC Genomics. 2009;10:142. doi: 10.1186/1471-2164-10-142 19335898 PMC2676302

[75] Guo B, Greenwood PL, Cafe LM, Zhou G, Zhang W, Dalrymple BP. Transcriptome analysis of cattle muscle identifies potential markers for skeletal muscle growth rate and major cell types. BMC Genomics. 2015;16:177. doi: 10.1186/s12864-015-1403-x 25887672 PMC4364331

[76] Moriel P, Johnson SE, Vendramini JMB, Mercadante VRG, Hersom MJ, Arthington JD. Effects of calf weaning age and subsequent management system on growth and reproductive performance of beef heifers. J Anim Sci. 2014;92:3096–3107. doi: 10.2527/jas.2013-7389 24778338

[77] Moriel P, Johnson SE, Vendramini JMB, McCann MA, Gerrard DE, Mercadante VRG, et al. Effects of calf weaning age and subsequent management systems on growth performance and carcass characteristics of beef steers. J Anim Sci. 2014;92:3598–3609. doi: 10.2527/jas.2014-7751 24948652

[78] Arthington JD, Cooke RF, Maddock TD, Araujo DB, Moriel P, Dilorenzo N, et al. Effects of vaccination on the acute-phase protein response and measures of performance in growing beef calves. J Anim Sci. 2013;91:1831–1837. doi: 10.2527/jas.2012-5724 23345563

